# A design of resonant inelastic X-ray scattering (RIXS) spectrometer for spatial- and time-resolved spectroscopy

**DOI:** 10.1107/S1600577520004440

**Published:** 2020-04-16

**Authors:** Yi-De Chuang, Xuefei Feng, Per-Anders Glans-Suzuki, Wanli Yang, Howard Padmore, Jinghua Guo

**Affiliations:** a Advanced Light Source, Lawrence Berkeley National Laboratory, 1 Cyclotron Road, MS 6-2100, Berkeley, CA 94720, USA

**Keywords:** Wolter type 1 mirrors, soft X-ray spectrometer, resonant inelastic X-ray scattering spectroscopy

## Abstract

The optical design of a Hettrick–Underwood-style soft X-ray spectrometer with Wolter type 1 mirrors is presented. The spectrometer with a nominal length of 3.1 m can achieve a high resolving power (resolving power higher than 10000) in the soft X-ray regime when a small source beam (<3 µm in the grating dispersion direction) and small pixel detector (5 µm effective pixel size) are used. Adding Wolter mirrors to the spectrometer before its dispersive elements can realize the spatial imaging capability, which finds applications in the spectroscopic studies of spatially dependent electronic structures in tandem catalysts, heterostructures, *etc*. In the pump–probe experiments where the pump beam perturbs the materials followed by the time-delayed probe beam to reveal the transient evolution of electronic structures, the imaging capability of the Wolter mirrors can offer the pixel-equivalent femtosecond time delay between the pump and probe beams when their wavefronts are not collinear. In combination with some special sample handing systems, such as liquid jets and droplets, the imaging capability can also be used to study the time-dependent electronic structure of chemical transformation spanning multiple time domains from microseconds to nanoseconds. The proposed Wolter mirrors can also be adopted to the existing soft X-ray spectrometers that use the Hettrick–Underwood optical scheme, expanding their capabilities in materials research.

## Abstract 2

The optical design of a soft X-ray spectrometer that utilizes Wolter mirrors to enable imaging-mode spectroscopy with a beam-size independent spatial resolution in, for example, tandem catalysts in reaction is presented. When such a spectrometer is used in pump–probe experiments to study the transient electron dynamics, the imaging capability of the Wolter mirrors can offer femtosecond time delay between the pump and probe beams with non-collinear wavefronts.

## 1. Introduction   

With the advent of third-generation synchrotron facilities that can deliver low emittance, high coherence, high brightness soft X-rays for materials research, some X-ray spectroscopies have undergone transformative changes over the past decades. One such technique is resonant inelastic soft X-ray scattering (RIXS) spectroscopy. In the RIXS process, the X-ray photons with energies tuned to the elemental absorption edges resonantly excite the core electrons to the unoccupied states, followed by the re-emission of lower energy photons when the core holes are filled by the electrons decaying from the occupied states (de Groot & Kotani, 2008 ▸). This coherent process couples to various elementary excitations whose nature is manifested by the electronic correlations. Since the dispersion relation of these excitations can be directly determined from the transferred photon energy and momentum, RIXS is thus an ideal technique for studying the electronic correlations that underpin the intriguing materials properties such as high temperature superconductivity, multiferroicity, quantum topological states, *etc*. (Kuiper *et al.*, 1998 ▸; Nordgren & Guo, 2000 ▸; Kotani & Shin, 2001 ▸; Schülke, 2007 ▸; Ament *et al.*, 2011 ▸; Simon & Schmitt, 2013 ▸; Schmitt *et al.*, 2014 ▸). RIXS is a second-order (two-photon) process with a very small cross section in the soft X-ray and EUV regimes; therefore, to make the measurements feasible within the limited experimental time, the throughput of the RIXS instrument was emphasized over its spectral resolution. But with the intense soft X-ray beam from undulators in the new generation of synchrotrons that can be tightly focused down to a few micrometres in size, high resolution RIXS spectroscopy in the soft X-ray regime became available about a decade ago and is now routinely performed to study the electronic structures and excitations of correlated, functional, and energy materials under UHV and even *in situ*/*operando* conditions (Ament *et al.*, 2011 ▸; Yang *et al.*, 2013 ▸; Liu *et al.*, 2014 ▸, 2015 ▸).

**Figure 1:**
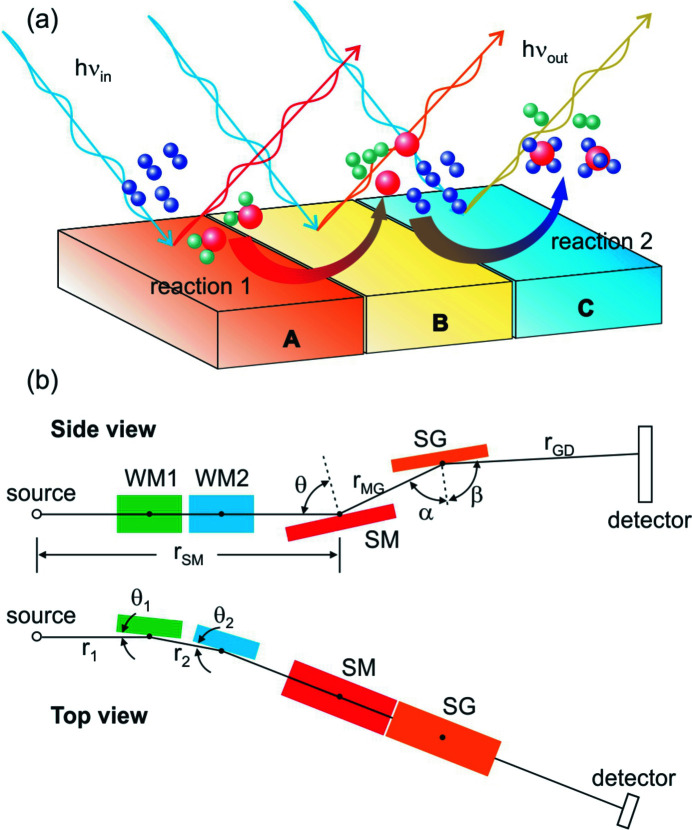
(*a*) Schematic illustration of a tandem catalyst consisting of three materials A, B, and C that are tailored for reactions 1 and 2. (*b*) Optical layout of proposed spectrometer. WM1, WM2, SM, and SG denote the Wolter mirror 1, Wolter mirror 2, spectrometer mirror, and spectrometer grating, respectively. *r*_SM_, *r*_MG_, and *r*_GD_ are the distances from source to SM1, SM1 to SG, and SG to detector, respectively. The incidence angle for SM is θ and the included angle for SG is α + β, where β is defined as a positive value. For imaging subassembly, *r*_1_ and *r*_2_ are the distances from source to WM1 and WM1 to WM2. The grazing incidence angles for WM1 and WM2 are θ_1_ and θ_2_.

The key instrument for soft X-ray RIXS spectroscopy is the grating-based spectrometer (referred to as the spectrometer hereafter) for analysing the energies of inelastically scattered X-rays from the sample. The grating inside the spectrometer disperses the X-rays with respect to their energies onto the imaging detector, while in the transverse direction the X-rays can either be collimated or focused to increase the angular acceptance of the instrument. These spectrometers, whether using spherical or plane gratings with constant or varied line spacing (VLS) rulings, can have disparate size and resolving power optimized for their science missions (Nordgren *et al.*, 1989 ▸; Hague *et al.*, 2005 ▸; Hatsui *et al.*, 2005 ▸; Chuang *et al.*, 2006 ▸, 2017 ▸; Ghiringhelli *et al.*, 2006 ▸; Agåker *et al.*, 2009 ▸; Fuchs *et al.*, 2009 ▸; Strocov *et al.*, 2010 ▸; Harada *et al.*, 2012 ▸; Yamane *et al.*, 2013 ▸; Chiuzbăian *et al.*, 2014 ▸; Lai *et al.*, 2014 ▸; Warwick *et al.*, 2014 ▸; Yin *et al.*, 2015 ▸; Dvorak *et al.*, 2016 ▸; Brookes *et al.*, 2018 ▸). However, using them to study the electronic structures of materials that are expected to exhibit strong spatial inhomogeneity can be challenging. For example, in tandem catalysts where multiple metal–metal-oxide interfaces tailored for different chemical reactions are assembled to perform multi-step reactions (Yamada *et al.*, 2011 ▸; Su *et al.*, 2016 ▸; Kim *et al.*, 2017 ▸; Xie *et al.*, 2017 ▸), the electronic structures of reaction sites will depend on their distances to the respective interfaces [see the schematic illustration of such tandem catalyst in Fig. 1(*a*) ▸]. In the case of CO_2_ methanation using Co nanocatalysts, the reduction of catalysts by hydrogen produced from the nearby Pt nanoparticles is expected to depend on the distance between Co and Pt nanoparticles (Beaumont *et al.*, 2014 ▸). The traditional way of scanning tandem catalysts across the focused X-ray beam and measuring the electronic structures during the catalytic reactions can only offer the ‘snap-shot’ view of each reaction site in this dynamic process. Furthermore, to avoid averaging spectra from vastly different reaction sites, the X-ray beam size needs to be extremely small, presumably on the order of 100 nm. Such a small X-ray beam can have extremely high fluence to induce non-linear or even sample heating/damage effects (Bostedt *et al.*, 2016 ▸; Wallander & Wallentin, 2017 ▸; Warren *et al.*, 2019 ▸). Mitigating these effects by reducing the X-ray flux density will lead to an excessively long measurement time, making the experiments impractical.

Such caveat can be circumvented if the spectrometer can differentiate the X-rays emitted from different reaction sites. This capability requires an additional spatial imaging component in the spectrometer so that the position information of source points can be relayed onto the detector. The optical design of such X-ray mirrors was pioneered by H. Wolter more than 60 years ago (Wolter, 1952*a* ▸,*b* ▸; Saha, 1987 ▸) and various types of Wolter X-ray mirrors have been used in X-ray telescopes (see http://chandra.harvard.edu/) and microscopes (Matsuyama *et al.*, 2010 ▸). Using Wolter mirrors in a cross-dispersion configuration was recently proposed to enable the recording of RIXS maps in the single acquisition (Warwick *et al.*, 2014 ▸). Following that approach, we will show that the Hettrick–Underwood-style soft X-ray spectrometer using a spherical mirror, instead of an elliptical cylindrical mirror, can also benefit from the Wolter mirrors (Hettrick & Underwood, 1986 ▸). The spectrometer will have dispersive and imaging subassemblies that will be separately described in this paper. Besides energy materials research, in ultrafast pump–probe RIXS spectroscopy the imaging capability of Wolter mirrors can offer pixel-equivalent femtosecond temporal resolution with non-collinear pump and probe beams.

## 2. Spectrometer optical design: dispersive subassembly   

### 2.1. Optical parameters   

The optical design presented in this paper is for a spectrometer that will be used in the AMBER (Advanced Materials Beamline for Energy Research) beamline at the Advanced Light Source (ALS), Lawrence Berkeley National Laboratory. The AMBER beamline has a dedicated Kirkpatrick–Baez (KB) mirror pair with vertical and horizontal demagnifications of 4.74 and 20, respectively. When operating the beamline at 10 µm exit slit setting to keep the resolving power higher than 10000 (or 30000 if high line density gratings with larger *c*_ff_ values are used), the vertical beam size on the sample is expected to be 2.1 µm. The spectrometer will be mounted at 90° scattering angle in the horizontal scattering plane with two gratings dispersing the scattered X-rays vertically to take advantage of this small beam size. A commercial CCD detector with 27 µm effective pixel resolution (with two-pixel point spread function) will be used in the spectrometer on day 1; however, the small pixel detector with ∼5 µm effective pixel resolution will be available in the future (Tremsin *et al.*, 2015 ▸; Andresen *et al.*, 2017 ▸). In that regard, the performance requirement for the spectrometer is set to have >5000 resolving power at C and O *K*-edges with 5 µm source and 27 µm detector pixel resolutions. To ensure that the optical design can achieve an even higher resolving power when small pixel detectors become available, the slope errors of optics need to be small. We have acquired high quality substrates for this spectrometer: 0.3 µrad RMS slope error for the spherical mirror (Winlight X) with a measured meridian radius of *R* = 2438.3 cm (designed meridian radius is 2435.5 cm ± 1% and 0.3 µrad RMS slope error over three tangential traces) and 0.15 µrad RMS slope error plane grating substrates (Insync, nominal flat figure with at most >10 km residual spherical radius removal to meet the 0.25 µrad RMS slope error requirement over three tangential traces). Even higher quality optics can be purchased from vendors like JTEC that utilizes the ultra-high-precision elastic emission machining (EEM) technology.

Two gratings are used to cover the operating photon energy range from 250 eV to 1500 eV. The gratings will be operated in outside (−1) order with nearly constant included angle. The low energy (LEG) and high energy (HEG) gratings will cover the photon energy range from 250 eV to 700 eV and 500 eV to 1500 eV, respectively. They are designed to completely correct the aberrations at 300 eV and 600 eV target photon energies with their central line densities and VLS terms scaled by a factor of two. One can also prescribe the VLS parameters specifically for C and O *K*-edges for LEG and HEG, respectively (see later discussion). Per our previous effort in developing the modular X-ray spectrometers (MXS) at the ALS, we intend to use nearly identical mechanical components for the optics chamber (Chuang *et al.*, 2017 ▸). This requirement sets the dimension of optics and the mounting scheme. In addition, limited by the space around the beamline and endstation area, the overall length of the spectrometer cannot exceed 3.5 m. With these considerations, we choose the nominal length of the spectrometer to be 3.1 m (distance measured from sample to the detector sensor).

The optical layout of the spectrometer is illustrated in Fig. 1(*b*) ▸. Like in the case of MXS, we follow the approach by Amemiya *et al.* (1996 ▸) and Amemiya & Ohta (2004 ▸) to analytically determine the VLS parameters for LEG. These parameters, adjusted to work with the measured 2438.3 cm meridian radius, are summarized in Table 1 ▸. In this table, *r*_SM_, *r*_MG_, and *r*_GD_ are the distances from sample (source) to the spectrometer mirror (SM), SM to the spectrometer gratings (SG), and SG to the detector. θ, α, and β are the incidence angle of SM, incidence and exit angles of SG, respectively. The included angle of SG is α + β, where β is defined as a positive value. This included angle will be changed slightly with respect to the photon energies to minimize the aberrations (see later discussion). *g*_0_, *g*_1_, *g*_2_, and *g*_3_ are the constant, linear, quadratic, and cubic terms in the VLS prescription: *g*(ω) = *g*_0_ + *g*_1_ω + g_2_ω^2^ + *g*_3_ω^3^, where *g*(ω) is the local groove density and ω is the signed tangential distance from the grating pole on the grating surface. We adopt the *SHADOW* convention such that the positive ω points to the downstream direction.

### 2.2. *SHADOW* simulations confirming the resolving power   

**Figure 2:**
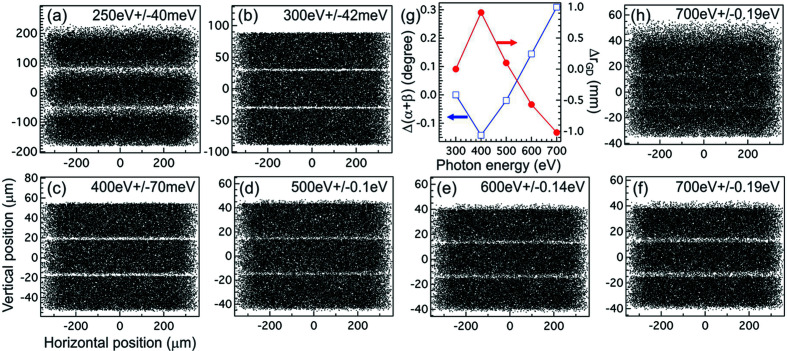
*SHADOW* simulations for LEG showing the images at the detector plane at selected photon energies: (*a*) 250 eV, (*b*) 300 eV, (*c*) 400 eV, (*d*) 500 eV, (*e*) 600 eV, and (*f*) 700 eV. In each figure, three photon energies are used for simulation with energy detuning listed on the top. (*g*) The amount of changes in included angle α + β (open blue squares, left axis) and the grating-detector distance *r*_GD_ (red filled circles, right axis) with minimization of vector sum of aberrations F_30_ and F_40_. (*h*) Simulation showing the effect without minimizing the vector sum of aberrations at 700 eV.

The performance of the dispersive subassembly of this spectrometer is simulated using *SHADOW* (Sanchez del Rio *et al.*, 2011 ▸). The results for LEG at selected photon energies are summarized in Figs. 2 ▸(*a*)–2(*f*) [for HEG, the resolving power should be the same as that of the LEG at scaled photon energies if the slope error contributions are not considered]. In each figure, rays with three different energies are propagated through the optical system and projected onto the detector plane normal to the optical principal axis. The central ray energy and the energy detuning are listed in each figure. In the simulations, the source is a 5 µm (H) × 100 µm (W) rectangle. The vertical beam divergence is set to 4.5 mrad, matching the 80 mm (L) × 80 mm (W) clear aperture of SM. The ruling area of SG is 80 mm (L) × 35 mm (W), so the horizontal beam divergence will be limited by the detector width unless it exceeds 110 mm. The commercial CCD detector with 27 mm square sensor will have a horizontal acceptance of 8.7 mrad; but, for the custom detectors, this acceptance angle can be even larger. To simplify the simulations, the horizontal beam divergence is limited to ±100 µrad. We also do not include the slope error contributions from SM and SG in the simulations because they are expected to be negligible due to the relatively large source size.

From these figures, we see that the spectrometer can achieve >5000 resolving power below 500 eV, meeting the design requirement. The resolving power increases monotonically with decreasing the photon energy until below 300 eV where the aberrations start to dominate. Even if the slope error contributions from SM and SG are included, the resolving power of LEG will still be higher than 5000 at 285 eV due to the source-limited resolution at the low energy end. For HEG, since it has the scaled VLS prescription *g*(ω) as the LEG, it is expected to have >5000 resolving power at 540 eV as well.

When photon energies are tuned away from the target energies (300 eV for LEG and 600 eV for HEG), we can perform additional optimization by slightly varying the grating included angle (α + β) to minimize the vector sum of the coma F_30_ and spherical aberration F_40_ terms while keeping the detector in focus (F_20_ = 0). This optimization procedure only works for photon energies above 300 eV because it yields β > 90° below this energy. As shown in Fig. 2(*g*) ▸, the procedure leads to a <0.3° change in the included angle (open squares, left axis) and ∼1 mm change in *r*_GD_ (filled circles, right axis). Such small changes can be easily accommodated by motorized grating rotation and detector translation (see later discussion). With this optimization, the spectral quality can be significantly improved. This is particularly important for photon energies that are far away from the target energies, such as in the case of 700 eV shown in Figs. 2(*f*) (with optimization) and 2(*h*) ▸ (without optimization) for the LEG. The aforementioned optimization procedure is not unique to the Hettrick–Underwood optical scheme because a similar procedure was proposed and used in high resolution RIXS spectrometers with VLS spherical gratings (Strocov *et al.*, 2011 ▸).

**Figure 3:**
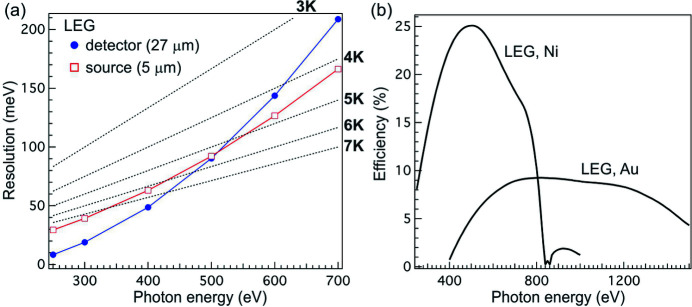
(*a*) 5 µm source (red open squares) and 27 µm detector pixel (blue filled circles) limited resolutions for LEG. The dashed lines mark the corresponding resolving power. (*b*) Calculated efficiency for LEG and HEG with Ni and Au coatings, respectively.

In Fig. 3(*a*) ▸, we show the source (red open squares) and detector pixel (blue filled circles) limited resolutions. These two contributions are not balanced at 300 eV; instead, they are balanced around 500 eV: below it, the energy resolution is dominated by the source size whereas, above it, it is dominated by the detector pixel size. This plot implies that even without improving the detector pixel resolution the resolving power of the spectrometer can still be increased by closing down the beamline exit slit at the expense of photon flux. We point out that the target photon energy is intentionally chosen below 500 eV (1000 eV) for LEG (HEG) to achieve better optimization on the vector sum of F_30_ and F_40_, thereby extending the operating photon energy range with good spectral quality.

### 2.3. Grating efficiency   

The grating efficiency calculated using *GSolver* is shown in Fig. 3(*b*) ▸. In the calculation, we have assumed the blazed profile for both gratings and the apex angle is set to 165°. The LEG will have an Ni coating to enhance the efficiency below 800 eV and the HEG will have an Au coating to avoid the elemental absorption edges below 1500 eV. The LEG and HEG will have blazed angles of 1.2° and 1.6°, respectively. The calculation shows that with these blazed angles the grating efficiency will be close to 25% at 500 eV and remains above 7% below 1200 eV. In addition, there is a crossover region between 500 eV and 800 eV where one can choose either the LEG or HEG for high efficiency or high resolution mode of operation. The acquired gratings from the vendor (Inprentus) have been measured and the peak efficiency for LEG (HEG) is 15% (6%) at 500 eV (800 eV). The reduction in efficiency can be traced to the variation and imperfection in the blazed profile over the ruled area, which can be simulated with *GSolver* by considering the contributions from more than ten randomly sampled local blazed profiles.

### 2.4. Operations with a fixed focal distance *r*_GD_   

**Figure 4:**
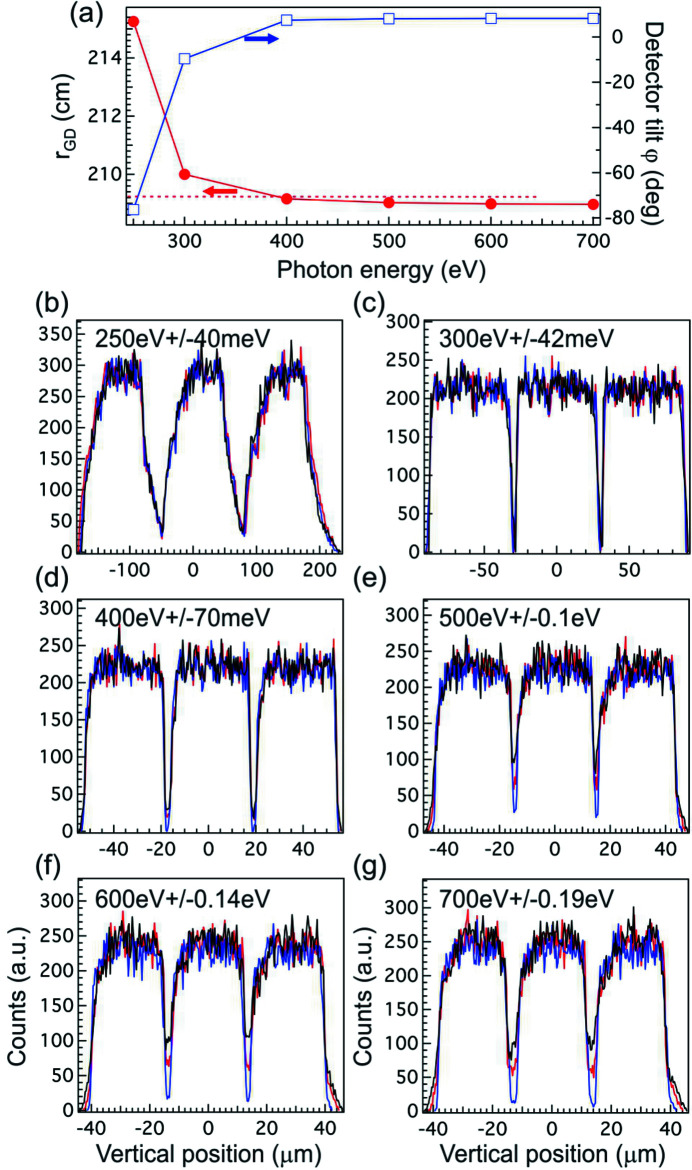
(*a*) *r*_GD_ (red filled circles, left axis) and detector tilt angle φ (blue open squares, right axis) as a function of photon energy. The red dotted line marks the fixed *r*_GD_ value. (*b*)–(*g*) Histograms produced by binning the images at the detector plane across the non-dispersive direction at (*b*) 250 eV, (*c*) 300 eV, (*d*) 400 eV, (*e*) 500 eV, (*f*) 600 eV, and (*g*) 700 eV. In each figure, the central ray photon energy and energy detuning are listed on the top. Red, black, and blue lines denote the results with nominal *r*_GD_, fixed *r*_GD_ = 209.224 cm without and with tweaking the spectrometer mirror angle θ, respectively.

The advantage of using the Hettrick–Underwood optical scheme is that the range of detector translation along the optical principal axis can be much smaller compared with the scheme that uses VLS spherical gratings. In Fig. 4(*a*) ▸, we show the *r*_GD_ as a function of photon energy (red filled circles, left axis). From this figure, we can see that *r*_GD_ only increases by 1 cm when the photon energy is changed from 700 eV to 300 eV; but, due to approaching the horizon energy (193 eV), *r*_GD_ increases rapidly below 300 eV and reaches 215.26 cm at 250 eV. The focal plane is not normal to the optical principal axis either. The detector canting angle φ relative to the optical principal axis can be calculated as follows: tan(φ) = δ*r*_GD_/(*r*_GD_δβ), where δ*r*_GD_ and δβ are evaluated through variation of the grating equation and the focus condition F_20_. The calculated φ angle [blue open squares, right axis in Fig. 4(*a*) ▸] changes sign in between 300 eV and 400 eV, and approaches a much larger value below 300 eV. One would typically implement some mechanism on the detector assembly to translate and tilt the detector to match the focal plane profile, and the required range of motion can be small: roughly 5 cm in translation and ±10° in tilt angle will be sufficient to match this focal plane.

However, these degrees of freedom will not be allowed when the Wolter mirrors are added into the spectrometer because they will lead to the defocusing for the Wolter mirrors and degrade the image quality (Warwick *et al.*, 2014 ▸). This statement is generic for spectrometers irrespective of which optical scheme is used; therefore, we need to evaluate the performance of the spectrometer with a fixed *r*_GD_. We set *r*_GD_ to 209.224 cm where the spectrometer is in focus at the zeroth order. In Figs. 4(*b*)–4(*g*) ▸, we show the histograms produced by binning the images on the detector plane across the non-dispersive direction (horizontally). The central ray energy and the energy detuning are listed on top of each figure, and three coloured lines represent three different simulation conditions. The red lines are just the histograms from images in Figs. 2(*a*)–2(*f*) ▸. The black lines are the results with *r*_GD_ = 209.224 cm after slightly varying the included angle of SG for optimization. As one can see, fixing *r*_GD_ does not change the full width at half-maximum (FWHM) of histograms except introducing spectral tails. These tails are negligible below 400 eV, but they become more pronounced at higher energies. The tail structure can be suppressed if one changes the incidence angle θ of SM, which is shown as blue lines in the figures. The amount Δθ is very small: about 0.00025° (4.3 µrad) per 100 eV from 300 eV, thus one only needs to change θ by 0.001° at 700 eV to achieve almost perfect focusing. In fact, the resulting image quality is even better than just optimizing the included angle of SG (red and black curves). This finding suggests that the Hettrick–Underwood-type spectrometer has enough flexibility to be operated with fixed focal length if both incidence angle θ of SM and the included angle (α + β) of SG can be tuned accordingly.

Before discussing the imaging subassembly, we would like to point out that one can also prescribe the VLS parameters specifically at 285 eV and 540 eV for LEG and HEG, respectively, to achieve the best performance at these two photon energies. The prescribed parameters are listed in parentheses in Table 1 ▸. Since these target photon energies are close to 300 eV and 600 eV, we expect to see very similar performance in between these two sets of parameters.

## 3. Spectrometer optical design: imaging subassembly   

Knowing that the Hettrick–Underwood-style spectrometer can be operated with a fixed *r*_GD_, we now look at the design of Wolter mirrors that can be installed before SM. The first and second Wolter mirrors, WM1 and WM2, will be placed at a distance *r*_1_ from the source and *r*_2_ from WM1, respectively [see the optical layout in Fig. 1(*b*) ▸]. They have grazing incidence angles θ_1_ and θ_2_ and they will be mounted sideways to provide horizontal imaging capability. Following Warwick *et al.* (2014 ▸), WM1 and WM2 will have hyperbolic cylindrical and elliptical cylindrical profiles, respectively, and will be arranged in the U configuration. With the parameters listed in Table 2 ▸, the combined magnification for the Wolter mirror pair is ∼ 20.

### 3.1. *SHADOW* simulations confirming the imaging capability   

**Figure 5:**
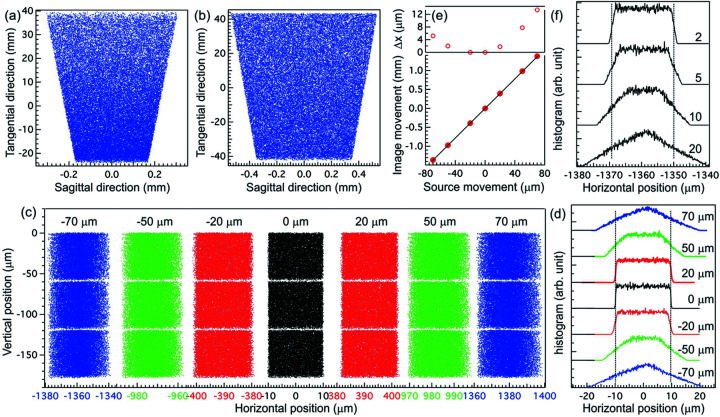
(*a*) Footprint of WM1. (*b*) Footprint of WM2. (*c*) Images at the detector plane from a 5 µm (V) × 1 µm (H) source with horizontal source movement listed in the figure. The incident photon energies are 300 eV ± 42 meV. The horizontal beam divergence is ±10 mrad. The incident angle θ of SM is changed by 0.0002° to optimize the image quality at the fixed *r*_GD_ = 209.224 cm. (*d*) Horizontal histograms [bin the image in panel (*c*) vertically] showing the changes in image profile with respect to the source movement listed in the figure. The histograms are shifted horizontally for comparison and offset vertically for clarity. (*e*) Bottom: the amount of translation used in aligning the histograms in panel (*d*) (red filled circles) versus the linear dispersion described in the text. Top: difference between the translation of histograms (red filled circles) and the linear dispersion (black line). (*f*) Horizontal histograms showing the change of image profile with respect to the angular acceptance of Wolter mirrors at −70 µm source movement. The numbers listed in the figure are the source divergence in mrad. Dashed lines in panels (*d*) and (*f*) denote the FWHM of the histograms.

The performance of the entire spectrometer is simulated using *SHADOW* again and the results are summarized in Fig. 5 ▸. The source is a 5 µm (H) × 100 µm (W) rectangle, but the beam divergence is set to 4.5 mrad (V) × 20 mrad (H). The horizontal beam divergence is much larger than the one used in the cross-dispersion RIXS setup (Warwick *et al.*, 2014 ▸) with a compromise in the field of view (see later discussion). Compared with conventional RIXS spectrometers, the horizontal acceptance angle needs to be large in order to compensate the intensity loss from two additional reflections. X-rays with three energies 300 eV ± 42 meV are propagated through the spectrometer to evaluate the resolving power with the addition of Wolter mirrors. As mentioned earlier, the incidence angle θ of SM will be slightly adjusted to optimize the image quality at a fixed *r*_GD_ = 209.224 cm. The footprints on WM1 and WM2 are shown in Figs. 5(*a*) and 5 ▸(*b*) ▸, respectively. Because of the very small *r*_1_ and *r*_2_, the clear apertures for both mirrors can be very small: 64 mm (L) × 0.6 mm (W) for WM1 and 86 mm (L) × 1.2 mm (W) for WM2. Smaller clear apertures may help fabricate these highly eccentric mirrors.

To study the performance of Wolter mirrors with respect to the source position and width (or field of view, FOV), we look at the image of a 5 µm (H) × 1 µm (W) source on the detector displaced across the optical principal axis of WM1. The simulations are summarized in Fig. 5(*c*) ▸. Without the source movement (black dots), the 1 µm wide rectangular profile is correctly reimaged by Wolter mirrors to form a 19.5 µm wide rectangle on the detector. In the meantime, the dispersive subassembly retains the resolving power as expected [see Fig. 2(*b*) ▸ for comparison]. When the source movement is increased, the image quality degrades, showing the reduced intensity around the less well defined edges without loss of resolving power. In Fig. 5(*d*) ▸, we show the horizontal histograms produced by binning the images in Fig. 5(*c*) ▸ vertically (along the grating dispersive direction). In this figure, two vertical dashed lines are used to mark the FWHM (19.5 µm) of the black curve that has zero source movement. For comparison, coloured histograms are shifted to line up with the black curve and offset vertically for clarity. With increased source movement, the histogram profile changes from a rectangle (black curve) to a trapezoid (green curve) around ±50 µm movement, and then to the asymmetric triangle (blue curve) at ±70 µm movement. Despite the noticeable change in the profile, the broadening in FWHM is less than 10% even with ±70 µm source movement. This reduced FOV compared with the cross-dispersion RIXS setup (Warwick *et al.*, 2014 ▸) can be attributed to the larger magnification and the angular acceptance (see later discussion).

Fig. 5(*e*) ▸ shows the amount of translation applied to align the histograms in Fig. 5(*d*) ▸ (red filled circles), overlaid with a linear dispersion (thin line) with a slope of 19.5 (19.5 µm image movement per 1 µm source movement). In the top panel, we show their difference in units of µm. The amount of translation applied to the histograms in Fig. 5(*d*) ▸ is almost linear with respect to the source movement, with largest deviation about 13 µm at +70 µm source movement. This 13 µm deviation from linearity is approximately 70% of one image width. The plot suggests that the dispersion can be view as linear within this 140 µm FOV.

### 3.2. Imaging quality and FOV of Wolter mirror pair   

Based on the results in Figs. 5(*d*) and 5(*e*) ▸, one can assert that with ±20 µm source movement there is negligible degradation in the image quality. With ±50 µm source movement one expects that X-rays emitted from a 1 µm wide source point will have ∼50% unweighted contribution from the neighbouring source points (∼25% from each side). That fraction goes up to 100% at ±70 µm source movement. However, after weighting the contributions by evaluating the area of the trapezoid and the triangle outside the dashed lines, the fraction goes down to <15% and ∼25% for ±50 µm and ±70 µm source movement, respectively. In that regard, one can assert that the FOV of this Wolter mirror pair can be up to 100 µm with a good imaging quality.

The size of the FOV will depend on the acceptance angle of the Wolter mirror pair: the larger the acceptance angle, the smaller the FOV becomes. This is shown in Fig. 5(*f*) ▸ where we compare histograms with different horizontal beam divergence at −70 µm source movement to simulate the masking of WM1. In this figure, the beam divergence is reduced from 20 mrad (bottom curve) to 10 mrad, 5 mrad, and 2 mrad (top curve). With reducing the beam divergence, the triangular profile evolves to trapezoidal and to almost rectangular, suggesting an improved image quality. With this evolution, one can say that the FOV can be increased by at least a factor of two with reduced acceptance angle down to 5 mrad; nevertheless, this implies a trade-off with the throughput.

In the previous simulations, we do not specify the spatial resolution of the Wolter mirror pair. The factor of ∼20 magnification cannot be extrapolated to infinitesimal length scales on the sample because the spatial resolution of the Wolter mirrors will be limited by the quality of the optics and the effective detector pixel size (without considering the challenging alignment of these mirrors). The slope errors of SM and SG will have a 1/cos(θ) forgiveness factor due to their sagittal arrangement, thus they are neglected in the estimation. With the state-of-the-art mirror fabrication capability, we expect to have <0.2 µrad RMS slope error for the highly eccentric aspheric mirrors. With these slope errors and without considering the system alignment, stability, and other environmental factors, the spatial resolution of Wolter mirrors will likely be around 100 nm. But to realize this resolution, the detector pixel size needs to be better than 2 µm in the current design (if considering the Nyquist limit with two pixels, the detector pixel resolution will need to be better than 1 µm).

### 3.3. Feasibility of the optical design   

As we discussed in the previous section, we have acquired high quality SM and SG substrates from commercial sources that meet the specifications, and even higher quality optics can be fabricated using current technology like EEM. Although we do not have the hyperbolic and elliptical cylinders for the imaging subassembly, we have learned from another project at the ALS (QERLIN, see Warwick *et al.*, 2014 ▸). The Wolter mirrors used in that project produce a smaller magnification and are less challenging to fabricate. The vendor (JTEC) has delivered these optics that meet the slope error requirement (<0.25 µrad RMS). The proprietary EEM method can in principle achieve the atomic layer-by-layer material removal, and, working closely with the metrology laboratory at the ALS (XROL) to produce even more challenging mirrors for ALS-U, we believe the proposed Wolter mirrors in Table 2 ▸ can be fabricated from the improved polishing technique out of such collaboration. If the mirror figures turn out to be slightly off from the designed profiles, the effect can be partly mitigated by slightly adjusting the mirror positions and changing their incidence angles (Yashchuk *et al.*, 2019 ▸).

The blazed gratings we have were produced using the AFM ruling technique pioneered by Inprentus Inc. The placement of grating grooves with this technique over a small length scale (hundreds of micrometres) is expected to have the AFM precision; however, such precision cannot be retained over a large length scale like the entire clear aperture. Presently, there is no reliable method to measure the groove placement precisely to evaluate the level of error; but, according to the vendor, the groove placement accuracy can be ensured to achieve a resolving power around 50000 over a 100 mm clear aperture, which is far better than what we designed for the spectrometer.

If one needs to achieve an even higher accuracy, one can resort to a proprietary grating fabrication technique that utilizes the commercial EUV lithography mask writer to produce the VLS groove pattern and then transfer that pattern onto a miscut Si(111) substrate to produce the blazed gratings. The process has been demonstrated to achieve ∼15 nm precision in the groove placement over a 120 mm clear aperture at high groove density (10000 lines mm^−1^). We have one such blazed VLS grating with 5000 lines mm^−1^ line density fabricated for the QERLIN project (Voronov *et al.*, 2017 ▸).

If one resorts to holographically ruled laminar gratings, there could be a larger discrepancy between the prescribed and fabricated VLS parameters. The Hettrick–Underwood optical scheme is quite powerful in compensating these errors compared with the VLS spherical grating scheme because it separates the tasks of focusing, energy monochromatization, and aberration correction into two optical elements; however, if the VLS *g*_2_ and *g*_3_ terms are off by 2% and 5% from the prescribed values, respectively (*g*_1_ can be largely corrected by translating the detector to vary the *r*_GD_ and is typically within <0.1% of the design value), it becomes challenging to recover the spectral resolution without reducing the angular acceptance (hence the throughput). Such caveat can be circumvented by close collaboration with vendors to change the VLS terms to globally reduce the spectral tail, and typical beamline monochromator gratings fabricated using such holographic ruling technique can achieve >20000 resolving power. Therefore, it is feasible to obtain the designed optics even with current state-of-the-art fabrication technologies. Besides, the proposed mechanical design will enable the combined motion, *i.e.* SM pitch angle and detector translation, to reduce the spectral broadening by incorrect VLS parameters. For imaging subassembly, translating/pitching WM1 and WM2 can be used to compensate the figure error as shown by Yashchuk *et al.* (2019 ▸).

### 3.4. Alignment tolerance, precision and the range of mechanical motion   

We have used *SHADOW* to simulate the effect of optical misalignment relative to the source (X-ray beam spot on the sample). To set the alignment tolerance for the dispersive subassembly, we require that the broadening in the FWHM of the histograms like those in Fig. 4 ▸ shall not exceed 10% or present noticeable spectral tail even if the broadening is less than 10%. For the imaging subassembly, we assume a full angular acceptance (20 mrad) in the simulation and look at the spatial profile of the histograms like those in Fig. 5(*d*) ▸. We set the criterion that the trapezoidal profile should not be worse than the green curves in Fig. 5(*d*) ▸. We also do not consider using the combined motion to increase the alignment tolerances as we reserve this option for correcting the errors in the optics figure and the grating ruling. The resulting numbers for alignment tolerance, the precision and range of mechanical motion are summarized in Table 3 ▸.

For the stability requirement, we set it to be 10% of the alignment tolerance. Based on Table 3 ▸, the stability requirements are set to be 500 nm vertical and 200 nm transverse over the course of 24 h. The floor scan around the area of the spectrometer confirms the required stability and the FEA also validates the mechanical design for the optics chamber and granite base for the spectrometer to meet these requirements.

## 4. Notes on the applications   

Current *in situ/operando* soft X-ray spectroscopy techniques (XAS, XES, and RIXS) at the ALS already provide element-specific access to the local chemical states in liquids, gas-phase molecules, and at the liquid/solid and gas/solid interfaces during the catalytic and/or electrochemical reactions. The particular challenge, however, remains to be able to conduct the *in situ/operando* characterization of electronic structure and the control of, for example, charge transfer and electron flow in ‘real world’ systems such as the solid/gas and solid/liquid interfaces, while simultaneously probing the chemical transformations on multiple time and length scales. RIXS is a particularly powerful soft X-ray technique for studying elementary excitations, such as vibrational (at high energy resolution), *d*–*d* (*f*–*f*), and charge transfer excitations that are critical for energy-related materials and chemical functions. An extension of this technique to facilitate the spatially and temporally resolved measurements of electronic dynamics will open a new direction for research in materials science and chemical transformation.

### 4.1. Mitigating the sample heating issue   

The typical way of extending the spectroscopy into the spectromicroscopy is to use a highly focused, nanometre size beam for probing the samples to achieve the spatial resolution. Unfortunately, such an approach has witnessed various technical issues for today’s material studies. First, in the soft X-ray regime, the very large horizontal beam size in third-generation synchrotron storage rings will require the reflective focusing mirrors to have enormous demagnifications to focus the beam down to the submicrometre; thus instead of using mirrors, zone plates are often used for such tight focusing (Samson & Ederer, 2000 ▸). However, zone plates have very low transmission (∼1% transmission) that renders most spectroscopies with low cross section unsuitable for microscopic applications. Additionally, the limited sample manipulation space in a system based on a zone plate focusing makes it difficult to incorporate the versatile real-world sample environments for *in situ/operando* studies. Second, with diffraction-limited storage rings that will deliver fully coherent soft X-ray beam with nearly round profile, one can envision using highly demagnifying KB mirrors (horizontal demagnification on the order of 100) to accomplish this goal (for example, see Eriksson *et al.*, 2014 ▸ and the focused issue therein contained). The fully coherent soft X-rays also boost the zone-plate transmission.

However, the tightly focused X-ray beam can cause issues previously encountered in the free-electron laser facilities. One such issue is the very high X-ray fluence that can introduce non-linear effects to distort the electronic structures or even sample damaging, and the effect would be more noticeable with a longer pulse like that in the storage ring where the notion of ‘destruction after measurement’ no longer applies. In particular, RIXS is a photon-hungry technique such that the radiation sensitivity issue cannot be simply mitigated by reducing the photon flux. Although the exact X-ray fluence for causing these effects will be sample-dependent, it has been found recently that even some transition-metal oxide systems can suffer radiation damage in RIXS experiments with a relatively low soft X-ray dose and a much relaxed beam size (Lebens-Higgins *et al.*, 2019 ▸). In general, a value like 1 mJ cm^−2^ is a good number to go with (for example, see Gregoratti *et al.*, 2009 ▸; Wang *et al.*, 2012 ▸). For 1000 eV photons, this fluence is equivalent to a flux density of 6 × 10^12^ photons cm^−2^, or 6 × 10^4^ photons µm^−2^, per pulse. With a storage ring that operates at 500 MHz repetition rate, the flux density becomes 3 × 10^13^ photons s^−1^ µm^−2^. Although this value is at most an order of magnitude higher than what is available at an existing high-resolution beamline, with improved coating and mirror/grating fabrication technology a comparable flux density for new high-resolution beamlines can be envisioned. Without doubt, the situation becomes even more dire with the beam focused down to the sub-micrmetre in both dimensions. For those beamlines, focusing the beam spot down to 1 µm^2^ with reflective mirrors will reach this threshold flux density. The mere reason that this fluence issue is not encountered in most third-generation synchrotron facilities thus far is due to the very low efficiency zone plates used to produce the tightly focused X-ray beam.

A less addressed issue is the local average heating and the associated mechanical stress from the enormous power density. This issue, in fact, takes place at an even lower fluence. With the 1 keV X-ray beam that reaches the 1 mJ cm^−2^ threshold fluence per shot (3 × 10^13^ photons s^−1^) over the 1 µm^2^ area, the deposited power will be 5 mW and the associated power density will be 5 kW mm^−2^. The temperature rise can be roughly estimated using the following equation: 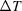 = 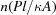, where *n* is a pre-factor depending on the geometry and is on the order of 1, κ is the thermal conductivity, *P* is the deposited power, *l* is the penetration depth, and *A* is the footprint of the beam. With the assumption of a 100 nm X-ray penetration depth and 1 µm^2^ area, the temperature rise Δ*T* will be around 50 K over the probe volume with κ = 10 W m^−1^ K^−1^ (typical for transition metal oxides). Although the average heating can lead to a considerable temperature rise on the sample when the X-ray beam is tightly focused down to the sub-micrometre level in size, the FEA shows that the transient heating will be even more severe and will follow the temporal profile of the X-ray beam that cannot be easily mitigated (Wallander & Wallentin, 2017 ▸). Further analysis will be required to investigate this issue for practical samples like quasi-two-dimensional oxides that host a variety of intriguing emerge physics phenomena.

But with the aforementioned spectrometer using the Wolter mirror pair for horizontal imaging, one does not have to tightly focus the beam horizontally. In fact, a wider beam spot that matches the FOV of Wolter mirrors will be more ideal. Use of a Wolter mirror pair not only avoids the high fluence high power density issue even when the vertical beam size is reduced down to ∼100 nm but also allows the spectromicroscopy to be implemented before the availability of the diffraction-limited storage ring.

### 4.2. Achieving femtosecond time resolution   

**Figure 6:**
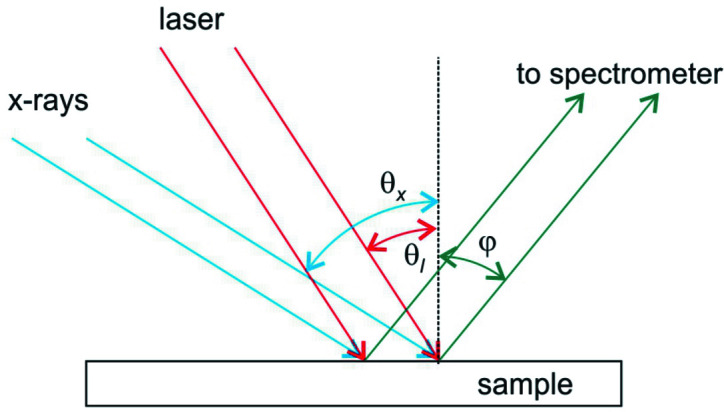
Schematic illustration of a pump–probe experiment setup. θ_*x*_, θ_l_, and φ are the incidence angles of X-ray (blue) and laser (red) beams onto the sample, and the emission angle to the spectrometer (green).

Besides using the spectrometer to study the spatially dependent electronic structures of materials, this spectrometer can also be used in pump–probe RIXS experiments. There, one can utilize the imaging capability of Wolter mirrors to achieve pixel-equivalent femtosecond time resolution. The idea is sketched in Fig. 6 ▸ and has been employed in the optical cross-correlation to mitigate the time jittering in free-electron lasers (for example, see Beye *et al.*, 2012 ▸). In this figure, θ_*x*_ and θ_*l*_ are the incidence angles of the probe X-ray beam (blue lines) and the pump laser beam (red lines) relative to the sample surface normal. φ is the emission angle to the spectrometer (green lines). *L* is the characteristic length scale, which can be the spatial resolution or FOV of Wolter mirror pair (with sufficient detector pixel resolution).

From this figure, one can see that the time difference between the wavefronts of pump and probe beams on the sample as viewed by the spectrometer detector is [*L*/cos(φ)][sin(*θ_x_*) − sin(*θ_l_*)]/*c*, where *c* is the speed of light. If the pump and probe beams are collinear, θ_*x*_ = θ_*l*_, there will be no time difference across the horizontal direction on the detector plane. To achieve the best temporal resolution per pixel, cos(φ) needs to be close to 1; or, equivalently, the sample should be placed close to normal emission geometry. Assuming the spatial resolution of Wolter mirror pair is *L* = 250 nm (using the 5 µm detector pixels and 20× magnification), the pixel-equivalent temporal resolution is (0.83 fs) × [sin(θ_*x*_) − sin(*θ_*l*_*)]. Based on this equation, the worst temporal resolution per pixel will be 0.83 fs when pump and probe beams are almost normal to each other. Even if the spatial resolution of the Wolter mirrors is larger than 250 nm, which leads to a larger pre-factor than 0.83 fs, one still can adjust θ_*x*_ and θ_*l*_ (making them more collinear) to achieve sub-femtosecond temporal resolution. We would like to point out that synchronizing the pump and probe beams to achieve femtosecond time delay is by no means an easy task, and is subjected to the time jittering and drift over the acquisition time.

One may be interested in recording a larger time-delay window per acquisition with reduced temporal resolution to a few femtoseconds. In this scenario, *L* is the FOV of a Wolter mirror pair and can be as large as 100 µm (see previous discussion). If the probe X-ray beam is perpendicular to both pump laser beam and the spectrometer, then the available time delay window will be ∼(0.33 ps)/cos(φ). One can then increase φ to achieve a picosecond recordable time window.

### 4.3. Probing chemical transformation in space and time   

An increasing number of experiments are performed under *in situ* or *operando* conditions in order to study the fundamental mechanisms underlying the complex processes such as the electrochemical energy conversion in realistic working conditions. A fundamental obstacle, however, remains to bridge the spatial, temporal, and thermodynamic scales that are simultaneously relevant for the outcome of chemical reactions. Often chemistry is based on statistical processes. Fluctuations in large ensembles lead to local conditions under which, at any given time, a small number of molecules can undergo chemical transformations. However, these conditions are only met for a very short period of time and only in a few specific locations. It is then and there where one needs the best possible spectral, temporal, and spatial resolution in spectroscopy in order to capture the correlated intra- and inter-molecular dynamics that are at the heart of chemical transformations. The location and time, however, of statistical processes is, by definition, not known. The discrepancy between the spatiotemporal heterogeneity of chemical reactions and the tight spatiotemporal restrictions of virtually all probes for fundamental interactions is one of the biggest challenges in the study of ‘real-world chemistry.’

A key challenge in understanding the aqueous-phase chemical reactions of organic molecules is developing novel ways of observing the formation of reactive intermediates in real time. Fully characterizing the mass and electronic structure of transient intermediates formed in a bimolecular reaction is central for elucidating condensed-phase reaction mechanisms. Direct observations of these short-lived species remain an outstanding hurdle due to the finite mixing times of current macroscopic reaction vessels (*e.g.* stopped flow kinetics) that are often longer than the lifetime of key reactive intermediates. For example, key free radical intermediates (*e.g.* per­oxy and alk­oxy radicals) in the atmospheric degradation of organic material often have condensed-phase lifetimes that are microseconds or shorter.

**Figure 7:**
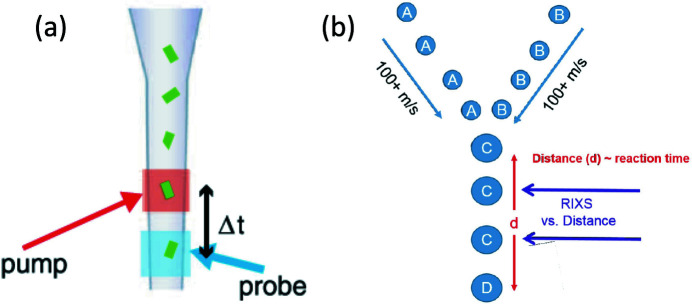
The schemes of RIXS sample systems: (*a*) the timing protocol consists of a fixed time of flight Δ*t* between the optical pump and X-ray probe beams (X-ray excitation and RIXS detection) (Kern *et al.*, 2013 ▸); (*b*) two droplets are levitated and rapidly mixed.

We envision, for slower bimolecular reactions, that the use of levitated liquid droplets may be advantageous for examining the millisecond and longer time scale kinetics. One scheme illustrated in Fig. 7(*a*) ▸ that enables the simultaneous X-ray diffraction and hard X-ray emission spectroscopy (at Mn *K*-edge) studies using the ultrashort, ultrabright FEL X-ray pulses was established to probe the intact atomic structure of PS II microcrystals and the intact electronic structure of its Mn_4_CaO_5_ cluster at room temperature (Kern *et al.*, 2013 ▸). The similar experimental method for a synchrotron based RIXS will help mitigate the soft X-ray photoreduction and radiation damage to the organic and biological samples. Another proposed experimental scheme could be the liquid jet or droplets levitation tools designed to access a broad range of reaction timescales when coupled to the X-ray beam. The time resolution of 1 ns can be achieved when the spatial resolution of 100 nm, with the help of Wolter mirrors in the spectrometer, is realized on a liquid jet at 100 m s^−1^ speed, so as to the time window of microseconds (over the 100 µm FOV of Wolter mirror pair) and multiple time domains can be readily obtained by adjusting the jet speed and the interaction point between the X-ray beam and the initial mixing location. For example, two droplets will be levitated and rapidly mixed using holographic optical tweezers as shown in Fig. 7(*b*) ▸. The reaction can be monitored both by the optical Raman spectroscopy and soft X-ray spectroscopy. This approach would foster the revolutionary opportunities to probe chemistry in merged trapped particles in the water window and above the carbon *K*-edge to achieve chemical contrast. The setup will provide information on compositional gradients and reaction intermediates within the liquid jet or droplets after they fuse and as the reaction progresses. The final droplet size would be changed to examine how greater degrees of confinement alter the chemistry.

## 5. Summary and conclusions   

We have presented an optical design of a Hettrick–Underwood-style spectrometer that uses a Wolter mirror pair consisting of a hyperbolic cylinder and an elliptical cylinder arranged in the U configuration for horizontal imaging. We show that this spectrometer can achieve a high resolving power (resolving power exceeding 10000) over the operating photon energy range from 250 eV to 1500 eV, if the small pixel detector and a small beamline exit slit setting are used. By tweaking the incidence angle θ of the spherical mirror and the included angle (α + β) of the VLS plane grating, the spectrometer can be operated with a fixed detector position *r*_GD_ without losing the spectral quality. By placing the Wolter mirrors close to the sample, a combined magnification of ∼20 can be achieved. With this magnification, the spatial resolution of the Wolter mirror pair can be around 250 nm with 5 µm detector pixel (or 500 nm considering the two-pixel Nyquist limit) and the FOV can exceed 100 µm with good imaging quality. This FOV can be further increased at the expense of throughput. For spectromicroscopy, the tightly focused X-ray beam can have fluence exceeding 1 mJ cm^−2^. This excessive local heating can take place at an even lower fluence and cause the non-linear effect, thermal inhomogeneity, and mechanical stress to distort the electronic structures of materials under study. The impact can be even greater with longer X-ray pulses like in the synchrotron storage ring where the thermal response can track the temporal profile of the X-ray beam. We show that the issues can be mitigated by using the Wolter mirror pair to allow the detector to perform the spatial imaging without the need of a tightly focusing the X-ray beam down to the sub-micrometre level in both dimensions. We argue that besides using the spectrometer to study the heterogeneous electronic structures of materials, the imaging capability of Wolter mirrors can offer the pixel-equivalent femtosecond temporal resolution in the pump–probe experiments and the spatial- and temporal-resolution at the nanometre and nanosecond, respectively, in the chemical reaction processes.

## Figures and Tables

**Table:** The VLS terms are defined as *g*(ω) = *g*_0_ + *g*_1_ω + *g*_2_ω^2^ + *g*_3_ω^3^, where ω is the signed distance to the grating pole along the tangential direction. Positive ω points to the downstream direction. Note that *R* is the measured value.

*r*_SM_ (cm)	*r*_MG_ (cm)	*r*_GD_ (cm)	θ (°)	α + β (°)	*n*	*R* (cm)
90	10	∼210	87	~ 173.5	−1	2438.3 (2435.5 ± 1%)

**Table:** 

VLS *g*(ω)	*g*_0_ (cm^−1^)	*g*_1_ (cm^−2^)	*g*_2_ (cm^−3^)	*g*_3_ (cm^−4^)	θ_B_ (°)	Coating
HEG, 600 eV	20000	190.7836 (0.1%)	7.9494 (2%)	−0.06133 (5%)	1.6	Au
(540 eV)	(20000)	(190.739)	(8.0949)	(−0.0716)		
LEG, 300 eV	10000	95.3918 (0.1%)	3.9747 (2%)	−0.03066 (5%)	1.2	Ni
(285 eV)	(10000)	(95.3811)	(4.0066)	(−0.03304)		

**Table:** 

*r*_1_ (cm)	*r*_2_ (cm)	θ_1_ (°)	θ_2_ (°)
10	10	2	2

**Table:** 

WM1, hyperbolic cylinder			WM2, elliptical cylinder		
*a* (cm), semi-major	*b* (cm), semi-minor	Eccentricity, ∊	*a* (cm), semi-major	*b* (cm), semi-minor	Eccentricity, ∊
5.508	0.505934	1.00420976	161.1615	3.3173	0.99978813

**Table 3:** Alignment tolerance, precision and range of mechanical motion Note that we adopt the *SHADOW* convention for the coordinates: on the optical surface, *X* is transverse to the beam path, *Y* is along the beam path, and *Z* is normal to the optical surface.

	Alignment tolerance	Mechanical motion (step resolution; range)
Spherical mirror		
MX (transverse)	±25 µm	N/A
MY (along beam path)	±25 µm	N/A
MZ (up/down)	±5 µm	N/A
MΘ_X_ (pitch)	±0.01°	0.0001°/step; 2°
MΘ_Y_ (roll)	±0.01°	N/A
MΘ_Z_ (yaw)	±0.1°	N/A

VLS plane grating		
GX (transverse)	N/A	10 µm/step; 40 mm
GY (along beam path)	±50 µm	N/A
GZ (up/down)	±10 µm	N/A
GΘ_X_ (pitch)	N/A	0.0001°/step; 5°
GΘ_Y_ (roll)	±0.01°	N/A
GΘ_Z_ (yaw)	±0.1°	N/A

Spectrometer		
SX (transverse)	±100 µm	N/A
SY (along the beam)	±100 µm	N/A
SZ (up/down)	±10 µm	N/A (or source vertical move)

Detector		
DY (along the beam)	N/A	1 µm/step; 150 mm

Wolter mirrors WM1		
WM1X	100 µm	N/A
WM1Y	±5 µm	0.5 µm/step; 1 mm
WM1Z	±2 µm	0.2 µm/step; 100 µm
WM1Θ_X_	±0.001°	0.0001°/step; 0.1°
WM1Θ_Y_	±0.01°	N/A
WM1Θ_Z_	±0.01°	N/A

Wolter mirrors WM2		
WM2X	100 µm	N/A
WM2Y	±10 µm	0.5 µm/step; 1 mm
WM2Z	±2 µm	0.2 µm/step; 100 µm
WM2Θ_X_	±0.001°	0.0001°/step; 0.1°
WM2Θ_Y_	±0.005°	N/A
WM2Θ_Z_	±0.01°	N/A
